# The impact of serum copper on the risk of epilepsy: a mendelian randomization study

**DOI:** 10.1186/s42494-023-00126-3

**Published:** 2023-06-25

**Authors:** Shihao Chen, Wenting Huang, Qi Xu, Tao He, Mulan Zhang, Huiqin Xu

**Affiliations:** Department of Neurology, The First Affiliated Hospital of Wenzhou Medical University, Wenzhou, 325000 China

**Keywords:** Serum copper, Epilepsy, Mendelian randomization, Genome-wide association studies, Single nucleotide polymorphisms

## Abstract

**Background:**

The relationship between serum copper and epilepsy has been elucidated in observational studies. In this study, we aimed to explore the causal relationship between serum copper and epilepsy using Mendelian randomization (MR) analysis.

**Methods:**

Single nucleotide polymorphisms (SNPs) associated with serum copper were used as instrumental variables for MR analysis to evaluate their causal effects on epilepsy. The main MR results were obtained by using the inverse variance weighting (IVW) method, supplemented by weighted median and MR-Egger regression. In addition, sensitivity analyses such as Cochran's *Q* test and pleiotropy test were used to assess these SNPs on epilepsy in terms of horizontal pleiotropy and heterogeneity.

**Results:**

The IVW method revealed that the serum copper was associated with an increased risk of generalized epilepsy (OR= 1.07; 95% CI 1.01- 1.14;* P* = 0.032), and the sensitivity analysis further supports the robustness of the results.

**Conclusions:**

The current study reveals a possible causal role for serum copper in increasing the risk of generalized epilepsy, which provide guidance for identifying potential risk factors for epilepsy.

**Supplementary Information:**

The online version contains supplementary material available at 10.1186/s42494-023-00126-3.

## Background

Epilepsy is a common neurological disorder that affects about 70 million people worldwide, with an incidence of 50.4 to 81.7 per 100,000 per year [1, 2]. The mechanisms of epileptic seizures are not fully understood. Some known causes of epilepsy include genetics, cerebrovascular disease, head injury, neurodegenerative diseases, etc. In recent years, researchers have extensively explored the relationship between trace elements and epilepsy [3]. Identifying this relationship can help discover potential risk factors and provide insight into epilepsy prevention and treatment.

Copper is an essential trace element in the human body, playing an important role in the development and function of the nervous system [4]. Copper is a vital trace element required by many enzymes in the brain. Administration of low doses of copper ion can induce epileptic seizures in animals by inhibiting Mg^2+^-ATPase and Na^+^-K^+^-ATPase, disrupting the Na^+^-K^+^ balance and leading to further epileptiform discharges [5, 6]. In addition, copper may produce toxic reactive oxygen species due to its redox activity, causing harm to the brain [7]. Both copper deficiency and excess can seriously affect brain function, leading to neurological diseases [8]. Studies have reported that elevated serum levels of copper may be associated with depression [9], Alzheimer's disease [10, 11], and multiple sclerosis [12]. Observational studies have suggested that elevated serum levels of copper may be associated with an increased risk of epilepsy [13], although another study did not find a correlation between them [14]. It is therefore essential to understand the causal relationship between serum copper and epilepsy.

Mendelian randomization (MR) analysis is a method that uses genetic variation in species as instrumental variables (IVs) to investigate the causality of an exposure on an outcome [15]. MR bears resemblance to randomized controlled studies in that it enables the investigation of causality by circumventing reverse causality and the influences of confounding factors [16]. Consequently, MR has emerged as a widely used epidemiological approach.

In the present study, we used genome-wide association study (GWAS) data on serum copper and epilepsy in an MR approach to further understand the risk factors influencing the development of epilepsy.

## Methods

### Study design

The MR analysis in this study was based on three key assumptions: (i) the selected genetic variants must be significantly associated with the exposure factor (serum copper); (ii) the selected genetic variants are not associated with other confounding factors; and (iii) the selected genetic variants affect the outcome (epilepsy) only through the exposure factor (serum copper) and do not affect the outcome through other pathways. If these three hypotheses hold, MR analysis could evaluate the causal relationship between the exposure factor and the outcome while avoiding potential confounders [15].

### Selection of instrumental variables

For serum copper, we used the large GWAS data from 2603 participants of European ancestry [17]. In this dataset, we used a criterion of *P* < 1 × 10^–5^ to screen for genetic variants significantly associated with serum copper and subsequently removed linkage disequilibrium (LD) between single nucleotide polymorphisms (SNPs) by *R*^2^ < 0.1. We also used an F-statistic to assess the reliability of the genetic variables, which was generally considered to have stronger instrument strength when *F* > 10 [18]. *R*^2^ was calculated as ${R}^{2}= 2 \times EAF \times (1-EAF) \times {Beta}^{2}$ [18], where Beta represents the estimated effect of genetic variation and EAF represents the effect allele frequency. *F* was calculated as $F= {R}^{2}\times (N-2)/(1-{R}^{2})$, where *R*^2^ was the cumulative proportion of variance in the phenotype explained by the SNPs included in the exposure factor, and *N* was the sample size.

Furthermore, we acquired comprehensive GWAS statistical data in association with epilepsy-related subtypes from the International League Against Epilepsy (ILAE). The sample sizes of the datasets are shown in Table 1. More detailed information on epilepsy cases can be found in the original study [19].

**Table 1:** Characteristics of included GWAS summary-level data of serum copper Levels and epilepsy

Trait	Consortium	Sample size	Population	Reference
Serum copper	-	2603	European	Hum Mol Genet. [17]
Epilepsy	ILAE		Mixed	Nat Com. [19]
Epilepsy (all documented cases)		44,889 (case: 15,212, control: 29,677)		
Generalized epilepsy (all documented cases)		33,446 (case: 3769, control: 29,677)		
Focal epilepsy (all documented cases)		39,348 (case: 9671, control: 29,677)		

*ILAE* the International League Against Epilepsy

### Statistical analysis

Three methods of MR were used in this study to analyze the causal effect of serum copper on epilepsy, including the inverse variance weighted (IVW) method, the MR-Egger regression method and the Weighted Median method. Each method uses a different hypothetical model to assess the causal effects and these are then used to check the robustness of the results. The IVW is considered the primary method of analysis as it is the gold standard for MR inference. It is primarily used for basic causal estimation and provides accurate results by calculating a weighted average of the Wald ratio estimates [20]. The MR-Egger method is used to detect sensitivities and it provides calculations after adjusting for pleiotropy [21]. Alternatively, in the median-weighted method, which allows for the presence of a 50% null of SNPs, the estimation of causal effects is then performed [22]. In addition, we also used Cochran's *Q* statistic to check the heterogeneity of the results in Heterogeneity test and performed a Pleiotropy test to check the polymorphism of results [21].

All analyses were conducted using the R software (version 4.1.3), with the MR analysis performed using the "TwoSampleMR" R package.

## Results

In the initial phase, we found that the serum copper was positively associated with the risk of generalized epilepsy (OR= 1.07; 95% CI 1.01–1.14; *P* = 0.032). In the sensitivity analysis of the significant findings, the Cochran's *Q* test indicated no heterogeneity between serum copper and generalized epilepsy (*Q* = 5.789; *P* = 0.447). Additionally, the polymorphism test demonstrated no evidence of cross-sectional polymorphism (Intercept = -0.006; *P* = 0.568) in the association between serum copper and generalized epilepsy. The MR estimates of genetically predisposed serum copper and susceptibility to epilepsy are presented in Table 2. The specific sensitivity analysis results are shown in Table 3.

**Table 2:** Causal effects of serum copper on the risk of epilepsy

Exposure	Outcome	Methods	SNP (*n*)	Beta	*P* value
serum copper	Epilepsy (all documented cases)	MR Egger	7	0.0738	0.2613
		Weighted median		0.0352	0.1482
		Inverse variance weighted		0.0402	0.0292
serum copper	Generalized epilepsy (all documented cases)	MR Egger	7	0.2107	0.0835
		Weighted median		0.1129	0.0082
		Inverse variance weighted		0.0704	0.0316
serum copper	Focal epilepsy (all documented cases)	MR Egger	8	0.0440	0.5906
		Weighted median		0.0315	0.2695
		Inverse variance weighted		0.0264	0.2454

*SNP* Single-nucleotide polymorphism, *Beta* the per-allele effect on cannabis use

**Table 3:** Evaluation of heterogeneity and pleiotropy using different methods

		Pleiotropy test			Heterogeneity test			
		MR-Egger			MR-Egger		IVW	
Exposure	Outcome	Intercept	SE	*P*	*Q*	*Q*_pval	*Q*	*Q*_pval
Serum copper	Epilepsy	-0.006	0.010	0.568	5.387	0.370	5.789	0.447
	Generalized epilepsy	-0.026	0.017	0.190	4.150	0.528	6.453	0.374
	Focal epilepsy	-0.003	0.013	0.818	8.461	0.206	8.542	0.287

*GWAS* Genome-Wide Association Studies, *SE* Standard error of Beta, *Q_pval* the *P*-value of Cochran’s *Q* test

**Fig. 1:**
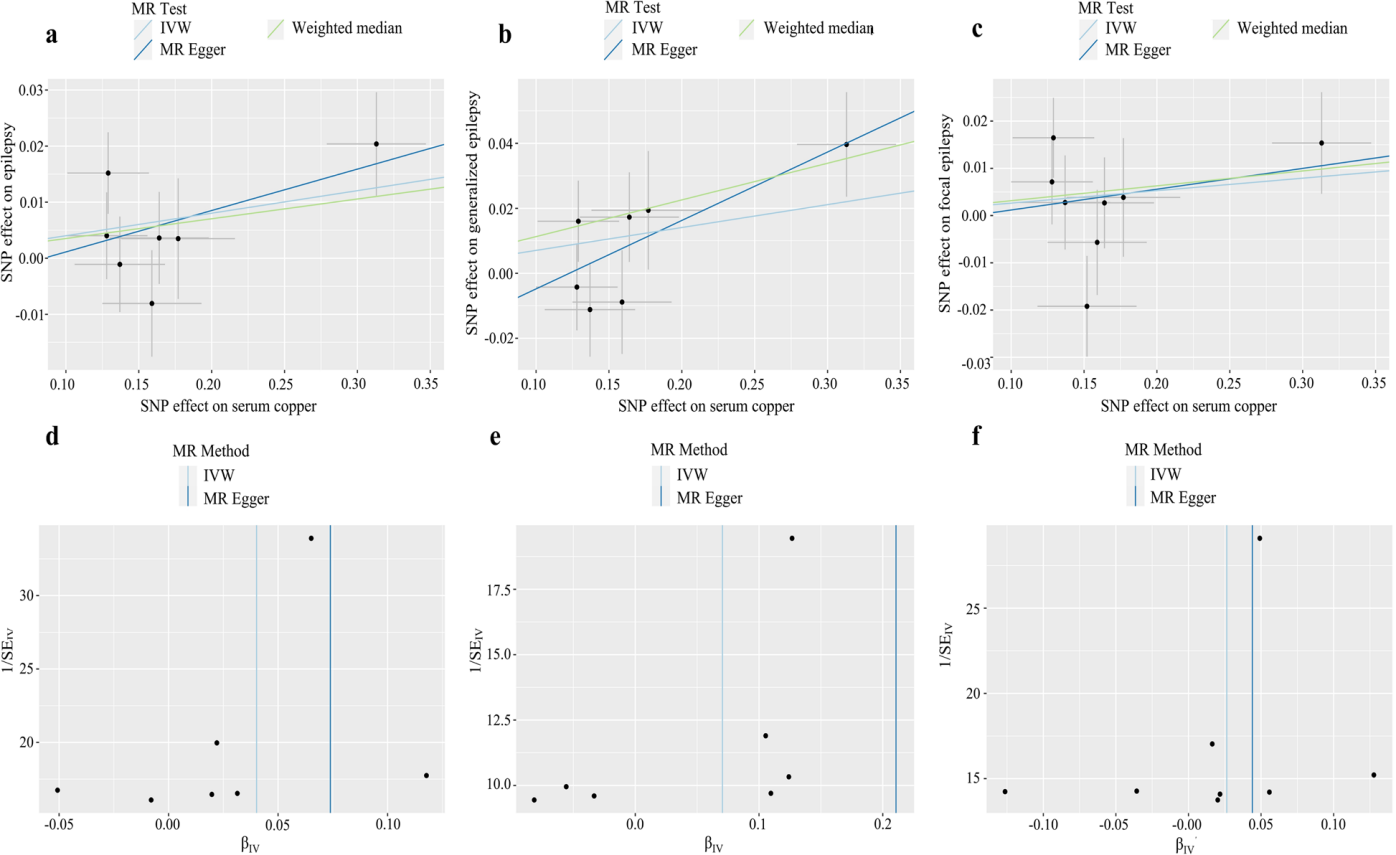
Scatter plots and funnel plots of MR analysis showed relationships between serum copper and epilepsy risk. **a**-**c** Scatter plots of relationships of serum copper with epilepsy (**a**), generalized epilepsy (**b**), and focal epilepsy (**c**). **d**-**f** Funnel plots of relationships of serum copper with epilepsy (**d**), generalized epilepsy (**e**), and focal epilepsy (**f**)

**Fig. 2:**
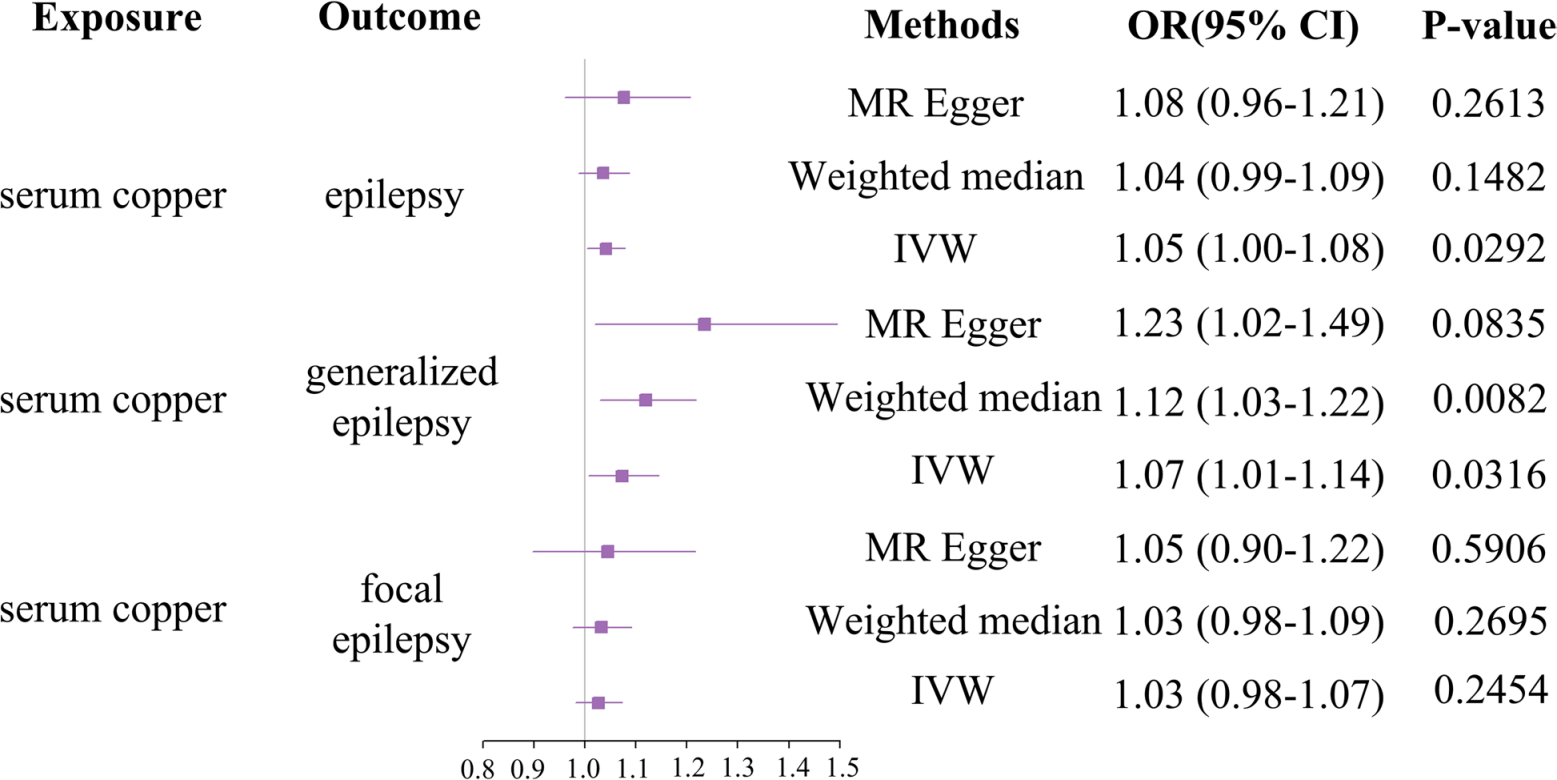
Forest plot of MR estimates of the causal effect of serum copper on epilepsy

Scatter plots and funnel plots of significant results are shown in Fig. 1. Forest plots of significant results are shown in Fig. 2.

Finally, detailed information on the SNPs involved in this study is given in Supplementary Tables S1, S2 and S3.

## Discussion

In this study, MR analysis showed that the elevated serum copper is associated with an increased risk of generalized epilepsy. However, no causal relationship was observed between serum copper level and focal epilepsy. Furthermore, we revealed no significant heterogeneity or evidence of horizontal pleiotropy in our results.

The copper content in the brain accounts for approximately 9% of the total copper in the human body, ranking the third highest concentration across organs [23]. Disruption of copper metabolism plays a vital role in the pathogenesis of epilepsy. Copper is also involved in the synthesis and release of neurotransmitters [24], regulating neuronal membrane stability and ion channels [25]. Additionally, it may impact the neuronal redox reactions and mitochondrial function. Mitochondria serve as the energy center of neurons, responsible for producing most of the cellular energy. Disturbances in copper metabolism can impair mitochondrial function, leading to disruptions in cellular energy metabolism, thereby affecting neuronal function [26, 27]. These changes may increase neuronal excitability, thereby elevating the risk of epilepsy. Studies have shown that exposure to copper induces oxidative stress and promotes apoptosis of HT22 mouse hippocampal neurons [28]. Furthermore, in recent years, cuproptosis is emerging as a novel mechanism of cell death. It has been demonstrated that copper ions can interact with sulfur transferase proteins in the tricarboxylic acid cycle, promoting abnormal oligomerization of these proteins. Moreover, copper ions can reduce the level of Fe-S cluster proteins, collectively triggering protein toxicity stress response that ultimately leads to cell death [29]. The association between cuproptosis and neurodegenerative disorders such as Alzheimer's disease has been extensively investigated [30, 31]. Wilson's disease, a genetic disorder of copper metabolism, leads to excessive accumulation of copper in the body, resulting in cuproptosis. High levels of copper can be observed in almost all brain regions of patients with Wilson's disease [32]. This condition is currently believed to be associated with seizures in epilepsy [33]. We hypothesize that cuproptosis may be one of the mechanisms underlying epilepsy, and our results also demonstrate that elevated serum copper levels increase the risk of generalized epilepsy.

Similar results have also been found in observational studies. Animal studies have shown that serum copper levels are significantly higher in dogs with controlled and uncontrolled epilepsy compared to normal or untreated dogs [34]. Furthermore, our research findings are consistent with a previous study involving 200 patients with genetic generalized epilepsy, which found a correlation between high serum copper levels and genetic generalized epilepsy [35]. Regarding the potential differences in the roles of copper ions in generalized epilepsy vs in focal epilepsy, it is worth noting that generalized epilepsy is a form of epilepsy that occurs widely throughout the brain, involving extensive neuronal networks [36]. Copper ions may disrupt neuronal function by modulating neuronal excitability and inhibitory pathways. Additionally, copper ions may interact with other ions, altering neuronal membrane potential and leading to abnormal discharges and seizure activity [25]. In focal epilepsy, abnormal discharges and seizures are primarily confined to specific brain regions. These seizures are often associated with specific lesions or injuries, such as brain tumors, infections, or trauma [37, 38]. Compared to generalized epilepsy, the disruption of neuronal networks is more localized in focal epilepsy. Therefore, copper ions may not have sufficient opportunity to enter or accumulate in the specific brain regions that affect seizure activity, resulting in a less significant impact on the occurrence and manifestation of focal epilepsy. However, it is important to note that these results are preliminary, and further research is needed to gain a deeper understanding of the relationship between copper and epilepsy.

This study has some advantages. First, our study was based on data of a large GWAS study provided by the ILAE Consortium, which includes a wide range of subtypes of epilepsy, contributing to a more comprehensive analysis of epilepsy. Second, we performed a sensitivity analysis to ensure that the results are not horizontally pleiotropic or heterogeneous. Finally, our MR analysis is superior to observational studies in that the SNPs were randomly assigned, leading to much less bias due to confounding factors.

However, this study also has certain limitations. First, the results of this study are based on European ancestry and it is unclear whether they can be generalised to other races. Second, the epilepsy dataset provided by the ILAE used in this study contained approximately 14% of cases of non-European descent. Therefore, population stratification is likely to introduce bias here.

## Conclusions

To conclude, this MR study offers genetic evidence supporting the association between elevated serum copper levels and an increased risk of generalized epilepsy, specifically within a European population. However, no such relationship was observed for focal epilepsy. These findings provide insights into the identification of potential risk factors of epilepsy. Nevertheless, further large-scale clinical studies are required to validate and expand these findings in the future.

## Supplementary Information

**Additional file 1: Supplementary Table S1.** Association strength of SNP allele frequencies and effect alleles with serum copper in outcome of epilepsy.

**Additional file 2: Supplementary Table S2.** Association strength of SNP allele frequencies and effect alleles with serum copper in outcome of generalized epilepsy.

**Additional file 3: Supplementary Table S3.** Association strength of SNP allele frequencies and effect alleles with serum copper in outcome of focal epilepsy.

## Data Availability

Data and materials related to the current study are openly accessible and available in the IEU Open GWAS Project repository (http://gwas.mrcieu.ac.uk), where they can be retrieved and utilized.

## Abbreviations

Beta: The per-allele effect on cannabis use
EA: Effect allele
EAF: Effect allele frequency
F: F statistic
GWAS: Genome-wide association study
ILAE: the International League Against Epilepsy
IVs: Instrumental variables
IVW: Inverse variance weighted
MR: Mendelian randomisation
OA: Other effect allele
*Q*_pval: The *P* value of Cochran's *Q* test
R^2^: Percentage of the variation explained by the SNP
SE: Standard error of Beta
SNPs: Single nucleotide polymorphisms
