# Supplementary material for: The impact of serum copper on the risk of epilepsy: a mendelian randomization study
Source: Acta Epileptol. 2023 Jun 25;5:15. doi: 10.1186/s42494-023-00126-3 (PMC11960368; doi:10.1186/s42494-023-00126-3)
Supplement: Supplementary file 3 — Additional file 3: Supplementary Table S3. Association strength of SNP allele frequencies and effect alleles with serum copper in outcome of focal epilepsy. [file 42494_2023_126_MOESM3_ESM.docx]

| Supplementary Table S3 \| Association strength of SNP allele frequencies and effect alleles with serum copper in outcome of focal epilepsy. | | | | | | | | | |  |
| --- | --- | --- | --- | --- | --- | --- | --- | --- | --- | --- |
| SNP | Chr | EA | OA | EAF | Beta | SE | *P*-value | *R*^2^ | *F* |  |
|  |  |  |  |  |  |  |  |  |  |  |
| rs10014072 | 4 | G | A | 0.672 | -0.164 | 0.034 | 1.13E-06 | 0.011857 | 472.1081 |  |
| rs12153606 | 5 | T | G | 0.1938 | -0.159 | 0.034 | 2.50E-06 | 0.0079 | 313.304 |  |
| rs2769264 | 1 | G | T | 0.161 | 0.313 | 0.034 | 2.63E-20 | 0.026467 | 1069.686 |  |
| rs3857536 | 6 | T | C | 0.5517 | -0.129 | 0.028 | 4.08E-06 | 0.008232 | 326.5664 |  |
| rs572585 | 2 | C | T | 0.7694 | -0.137 | 0.031 | 9.13E-06 | 0.00666 | 263.8064 |  |
| rs764560 | 7 | T | C | 0.3221 | -0.128 | 0.028 | 6.42E-06 | 0.007155 | 283.5472 |  |
| rs7206796 | 16 | T | C | 0.2197 | -0.152 | 0.034 | 9.26E-06 | 0.007922 | 314.169 |  |
| rs9324493 | 8 | G | A | 0.1431 | -0.177 | 0.039 | 5.94E-06 | 0.007683 | 304.6468 |  |
| SNP, single-nucleotide polymorphism; EA, effect allele; OA, other effect allele; EAF, effect allele frequency; R^2^, percentage of the variation explained by the SNP; F, F statistic; Beta, the per-allele effect on cannabis use; SE, standard error of Beta; *P*-value is for the genetic association. | | | | | | | | | |  |
